# Detection of *Legionella anisa* in Water from Hospital Dental Chair Units and Molecular Characterization by Whole-Genome Sequencing

**DOI:** 10.3390/microorganisms6030071

**Published:** 2018-07-18

**Authors:** Giuseppe Fleres, Natacha Couto, Mariette Lokate, Luc W. M. van der Sluis, Christophe Ginevra, Sophie Jarraud, Ruud H. Deurenberg, John W. Rossen, Silvia García-Cobos, Alex W. Friedrich

**Affiliations:** 1 University of Groningen, University Medical Center Groningen, Department of Medical Microbiology, 9713 GZ Groningen, The Netherlands; g.fleres@umcg.nl (G.F.); n.monge.gomes.do.couto@umcg.nl (N.C.); m.lokate@umcg.nl (M.L.); ruuddeurenberg@home.nl (R.H.D.); john.rossen@gmail.com (J.W.R.); alex.friedrich@umcg.nl (A.W.F.); 2 Center of Dentistry and Oral Hygiene, University Medical Center Groningen, 9712 CP Groningen, The Netherlands; l.w.m.van.der.sluis@umcg.nl; 3 International Center for Infectiology Research (CIRI), Inserm, U1111, CNRS, UMR5308, ENS de Lyon, Université Claude Bernard Lyon 1, 69007 Lyon, France; christophe.ginevra@univ-lyon1.fr (C.G.); sophie.jarraud@univ-lyon1.fr (S.J.); 4 Institute for Infectious Agents, National Reference Centre for Legionella, Groupement Hospitalier Nord, Hospices Civils de Lyon, 69007 Lyon, France

**Keywords:** *Legionella* spp., whole-genome sequencing (WGS), core-genome multi-locus sequence typing (cgMLST), whole-genome multi-locus sequence typing (wgMLST), next-generation sequencing (NGS)

## Abstract

This study aims to assess contamination with *Legionella* spp. in water from dental chair units (DCUs) of a hospital dental ward and to perform its molecular characterization by whole-genome sequencing (WGS). We collect eight water samples (250 mL) from four DCUs (sink and water-syringe). Samples are tested for the presence of *Legionella* spp. (CFUs/mL) by culturing according to the Nederland Norm (NEN) 6265. Three DCUs are found positive for *Legionella anisa*, and four isolates are cultured (sink *n* = 2, water-syringe *n* = 1; two isolates from the same chair) with 1 × 10^2^ CFU/mL. Whole-genome multi-locus sequence typing (wgMLST) results indicate that all strains belong to the same cluster with two to four allele differences. Classical culture combined with WGS allows the identification of a unique clone of *L. anisa* in several DCUs in the same hospital dental ward. This may indicate a common contamination source in the dental unit waterlines, which was fixed by replacing the chairs and main pipeline of the unit. Our results reveal tap water contamination in direct contact with patients and the usefulness of WGS to investigate bacterial molecular epidemiology.

## 1. Introduction

*Legionella* spp. are environmental Gram-negative bacteria, predominantly found in aquatic environments and water systems. They have been described as causative agents of a severe form of pneumonia called Legionnaires’ Disease (LD) or a milder, flu-like illness known as Pontiac fever. The genus *Legionella* includes more than 60 species, with *Legionella pneumophila* being the most common human pathogen, causing 90% of all outbreaks of LD [1]. Infection occurs when the microorganism in droplets enters the airway and encounters the alveolar macrophage in the distal airway. After uptake by the macrophage, *L. pneumophila* remodels its phagosome into a hospitable niche, the *Legionella*-containing vacuole (LCV) [2]. The ability to manipulate the host-cell processes is due to a large and versatile repertoire of effector proteins (~300 effectors in *L. pneumophila*) translocated from the LCV into the host cell cytosol using a type IV secretion system called Icm (intracellular multiplication) or Dot (defect in organelle trafficking) [3]. These effectors allow the LCV to escape the usual fate of a phagosome.

Next to the classical *L. pneumophila* serogroups, at least 20 species have been associated with human disease, particularly in immunocompromised hospitalized patients. *Legionella anisa*, the most common non*-pneumophila Legionella* species in the environment, was first isolated from an environmental sample of drinking and cooling water [4]. The role of *L. anisa* as a causative agent of LD and Pontiac fever has been previously demonstrated in several countries [5], and it may be hospital-acquired as previously described in reported cases of LD and pleural infection [6]. In addition, this bacterium has been found to cause extra pulmonary infections such as chronic endocarditis [7] and osteomyelitis [8]. The major route of transmission of *Legionella* spp. is through inhalation or aspiration of contaminated aerosols, which are highly produced in a dental unit environment [9]. The flow of water in the dental unit waterlines (DUWLs) is low, and the construction generally allows for the retention of water, resulting in the formation of biofilm and microbial growth on the inside of the pipes [10]. The instruments used in patient treatment form micro-aerosols, increasing the risk of infection for both patients and dental personnel. Although a direct link between the dental unit and the patients is rarely shown, the water delivered by the DUWLs has been shown to be one of many possible sources for *Legionella* infection [11]. Schönning et al. described one of the first cases of legionellosis acquired through a dental unit [11], highlighting the need to monitor water quality to protect patients and health-workers from acquiring *Legionella* infections. This study aimed to assess water contamination by *Legionella* spp. in dental chair units (DCUs) in a hospital dental ward and to determine its molecular characterization by whole-genome sequencing (WGS).

## 2. Materials and Methods

### 2.1. Sampling

**Figure 1:**
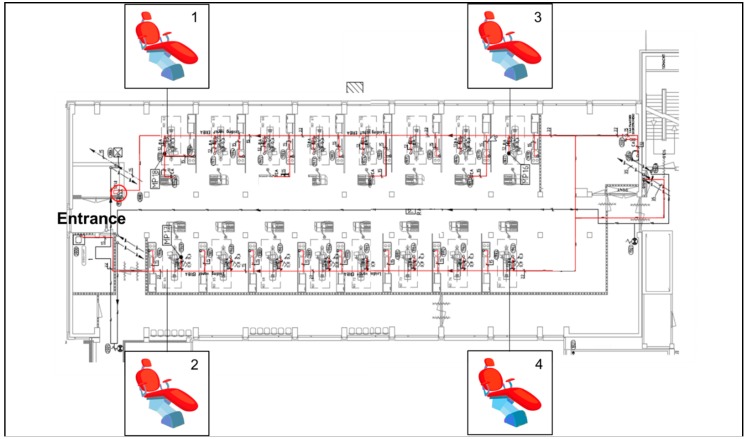
Dental unit waterline (DUWL) map; the main water pipeline is marked in red.

In June 2017, a total of eight water samples were collected from four DCUs located at the beginning and the end of a dental ward (Figure 1) at the Center of Dentistry and Oral Hygiene, University Medical Center Groningen (UMCG). Samples were taken at two different sites of the chairs, i.e., from the sink and the air-water syringe. Before the sampling, both water sources were rinsed for 10 s. The water was collected in sterile 250 mL glass bottles. All samples were maintained at isothermal conditions during transportation to the laboratory at the Medical Microbiology Department (UMCG) and processed within 24 h.

### 2.2. Culture and Species Identification

The water samples were analyzed for *Legionella* spp. by standard culture techniques according to the Dutch guideline or Nederland Norm (NEN) 6265, as follows: 250 mL of water sample were filtered through a polyether sulfone membrane with a porosity of 0.2 µm (Pall Life Sciences, Ann Arbor, MI, USA). The intact membranes were aseptically removed, placed into sterile 50 mL screw-capped tubes, and re-suspended in 10 mL of the original water samples. Each concentrated water sample was sonicated for five minutes to dislodge bacterial cells from the membranes. The cell suspension was placed in a 50 °C water bath for 30 min. The heat treatment of the concentrated water samples was used as a selective method to reduce the amount of non-*Legionella* bacteria. An aliquot of 100 µL was aerobically cultured on buffered charcoal yeast extract (BCYE) agar supplemented with cysteine (Oxoid ThermoScientific, Basingstoke, UK) for 7 days at 35 °C (±2 °C). Species identification was done using MALDI-TOF (Bruker, Daltonik Gmbh, Bremen, Germany). To confirm the previous identification, the OrthoANI algorithm was used to confirm species identification [12] by assessing the overall similarity between our isolates and six reference genomes downloaded from the NCBI genome database (http://www.ncbi.nlm.nih.gov/genome/): three *L. anisa* strains (strain Linanisette NZ_CANP00000000.1; strain FDAARGOS-200 NBTX00000000.2; strain WA-316-C3 NZ_LNXS00000000.1), one *Legionella dumoffii*, *Legionella longbeachae* strain NSW150, and *Legionella pneumophila* strain Philadelphia, which were also used for further comparative genomic analysis. OrthoANI values were obtained, and a phylogenetic tree was constructed using the orthologous average nucleotide identity tool.

### 2.3. Susceptibility Testing

Susceptibility testing was performed using E-test (BioMérieux, Marcy-l'Étoile, France) on BCYE-α (Oxoid ThermoScientific), as previously described [13]. Six antibiotics were tested: Azythromycin (AZI), Clarithromycin (CLA), Erythromycin (ERY), Moxifloxacin (MOX), Levofloxacin (LEV), and Doxycycline (DOX). The plates were incubated at 35 °C for 48 h before reading the minimum inhibitory concentration (MIC) value. Results were interpreted comparing the MIC of the isolates with the MIC distribution for *L. pneumophila*, according to the EUCAST guidance document on *Legionella* [13].

### 2.4. Short-Read Whole-Genome Sequencing

DNA extraction of *L. anisa* isolates was performed using the DNeasy UltraClean Microbial Kit (Qiagen, Hilden, Germany). The extracted DNA was diluted to 0.2 ng/µL, and 1 ng was used for the library preparation, using the Nextera XT Library Preparation kit (Illumina, San Diego, CA, USA) according to the manufacturer’s protocol. Cluster generation and sequencing were attained with a MiSeq Reagent Kit v2 500-cycles Paired-End in a MiSeq instrument (Illumina, San Diego, CA, USA).

### 2.5. Long-Read Whole-Genome Sequencing

One isolate (3A) was randomly selected for long-read sequencing to improve the quality of genome assemblies. The DNA libraries were prepared without shearing to maximize sequencing read length. The library for *L. anisa* was prepared using the 1D Ligation sequencing kit (SQK-LSK108) and the Native barcoding kit (EXP-NBD103) (Oxford Nanopore Technologies [ONT], Oxford, United Kingdom). The protocol for the 1D Ligation sequencing kit was followed as described by the manufacturer. The final library was loaded onto a FLO-MIN106 R9.4 flow cell. The run was performed on a MinION (Oxford Nanopore Technologies [ONT]) device using the NC_48Hr_Sequencing_Run_FLO-MIN106_SQKLSK108 protocol with 963 available pores (466, 320, 148 and 29 pores per group). The run proceeded for the full 48 hours.

### 2.6. Data Analysis

#### 2.6.1. Genome Assembly and Annotation

Illumina raw short-reads were checked for quality, trimmed and *de novo* assembled into contigs using CLC Genomics Workbench version 10 (CLC, QIAGEN, Aarhus, Denmark) using default settings. For MinION long-reads, base calling was performed using Albacore v1.2.2 (ONT), and data quality was analyzed through Poretools v0.6.0 [14]. Hybrid assemblies of short- and long-reads were performed using Unicycler v0.4.1 [15]. Bandage v0.8.1 [16] was used to visualize the assembly graphics. RAST v2.0 [17] was used to annotate the hybrid assembled genome.

#### 2.6.2. Construction of A Core- and Whole-Genome Multi Locus Sequence Typing (cgMLST/wgMLST) Using Publicly Available *L. anisa* Genomes

Since no cgMLST/wgMLST scheme was available for *Legionella anisa*, cgMLST and accessory genome schemes were constructed using the genomes of three *L. anisa* strains downloaded from NCBI (RefSeq: NZ_CANP00000000.1; RefSeq: NZ_NBTX00000000.1; RefSeq: NZ_LNXS00000000.1), using Ridom SeqSphere+ cgMLST Target Definer with the following parameters: A minimum length filter that removes all genes smaller than 50 bp; a start codon filter that discards all genes that contain no start codon at the beginning of the gene; a stop codon filter that discards all genes that contain no stop codon, that contain more than one stop codon, or that do not have the stop codon at the end of the gene; a homologous gene filter that discards all genes with fragments that occur in multiple copies within a genome (with identity of 90% and > 100 bp overlap); and a gene overlap filter that discards the shorter gene from the cgMLST scheme if the two genes affected overlap > 4 bp. The remaining genes were then used in a pairwise comparison using BLAST version 2.2.12 (parameters used were word size 11, mismatch penalty −1, match reward 1, gap open costs 5, and gap extension costs 2). All genes of the reference genome that were common in all query genomes with a sequence identity of ≥90% and 100% overlap and, with the default parameter stop codon percentage filter turned on, formed the final cgMLST scheme. The final wgMLST scheme consisted of 3140 core genes and 540 accessory genes (in total 3680 genes). The calculated distances were used for minimum spanning tree analysis using the parameters “pairwise ignoring missing values” during calculation.

#### 2.6.3. Comparative Genomic Analysis

The pan-genome of all isolates (*n* = 4) and *L. anisa* reference strains (*n* = 3) was inferred using Roary v3.8.0 [18] to investigate the *Legionella anisa* intra-species genome diversity. Genomes were annotated using Prokka v1.12. beta [19], and the annotations were provided to Roary as input; afterwards, Roary produced a gene presence/absence matrix. The graphic representation of this matrix was generated by a script which is a part of the Nullarbor pipeline (https://github.com/tseemann/nullarbor).

#### 2.6.4. Antibiotic Resistance Genes, Virulence Factors and Dot/Icm Effectors Detection

Antibiotic resistance genes (ARGs) and virulence factors (VFs) were detected from assemblies using ABRicate v0.7 (https://github.com/tseemann/abricate) and the CARD database, applying a cut-off value of ≥70% identity and ≥80% coverage. All the isolates were also screened for point mutation linked to resistance to macrolides (*L4*, *L22* and *23s* genes), fluoroquinolones (*gyrA, gyrB* and *parC* genes), and rifampicin (*rpoB* gene) using CLC Genomics Workbench version 10 [20,21,22]. In addition, SRST2 v0.2.0 [23] and an in-house database including all known *L. anisa* effector proteins (*n* = 130) [3] were used for the detection of *Dot/Icm* effectors. SnapGene v4.1.7 was used to visualize the annotated sequences.

#### 2.6.5. Plasmid Analysis

Fasta files of two complete plasmid sequences, named plasmid p3A1 (RefSeq: NZ_CP029564.1) and p3A2 (RefSeq: NZ_CP029565.1), were obtained from the Illumina-ONT hybrid assembly of isolate 3A (RefSeq: GCF_003176875.1). Sequences were initially aligned through BLASTN to the nucleotide collection of NCBI and then annotated using PATRIC [24]. The presence of p3A1 and p3A2 plasmids was investigated in a set of 49 *Legionella* spp. genomes database (8 *L. pneumophila* genomes and 41 non-*L. pneumophila* genomes) (the list of all *Legionella* strains and accession numbers is provided as supplementary material). For this purpose, SeqFindR was used to look for similar p3A1 and p3A2 plasmid features in the aforementioned genomes’ database. The phylogeny of the 49 *Legionella* spp. genomes was assessed by extracting the 16S rDNA region from isolates using an in-house script, and afterwards, a tree was generated using Phylogeny.fr [25].

## 3. Results

### 3.1. Legionella Isolation and Species Identification

**Figure 2:**
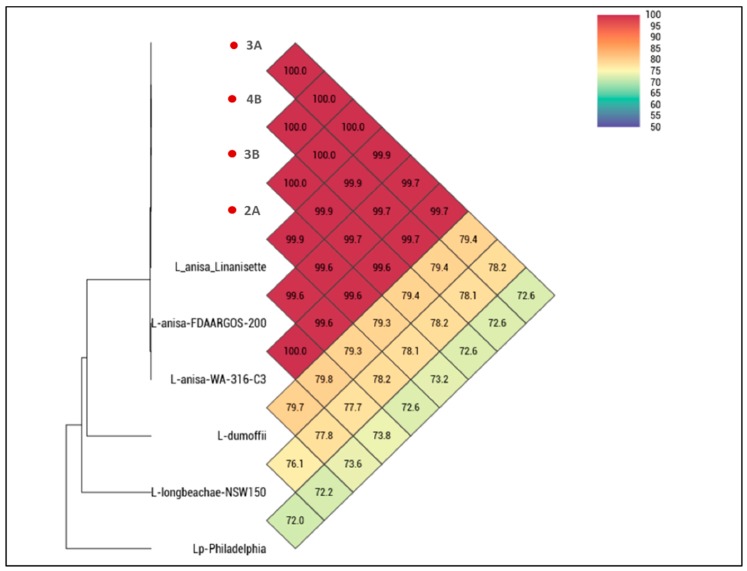
Heatmap generated with OrthoANI (Average Nucleotide Identity by Orthology) values calculated from OAT software. The isolates from this study are marked in red.

Three DCUs were positive for *Legionella* spp. (*n* = 2 sink, *n* = 1 water syringe) with 1 × 10^2^ CFU/mL and four isolates (chair-2 *n* = 1; chair-3 *n* = 2; chair-4 *n* = 1) were obtained and identified as *Legionella anisa.* The isolates were phenotypically susceptible to all antibiotics tested. The OrthoANI analysis showed 100% average nucleotide identity between our isolates (Figure 2). When comparing isolates from this study to all publicly available *L. anisa* reference genomes, values were between 99.9% (in the case of *L. anisa* strain Linanisette) and 99.6% for the other *L. anisa* strains. The comparison concerning our isolates and non-anisa *Legionella* spp. genomes showed always identity values below 80%.

### 3.2. wgMLST Analysis

**Figure 3:**
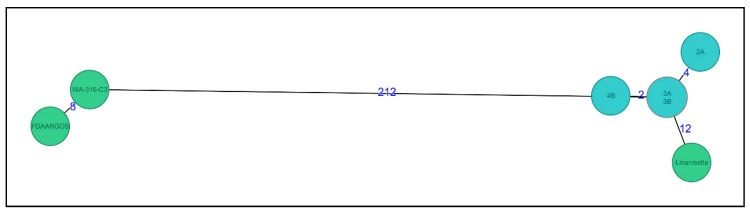
Minimum spanning tree of four *L. anisa* isolates (light blue) (named as 2A, 3A, 3B, and 4B) from dental chairs and three reference genomes (green) from NCBI database (RefSeq: NZ_CANP00000000.1; RefSeq: NZ_NBTX00000000.1; RefSeq: NZ_LNXS00000000.1) (named as Linanisette, FDARGOS_200, and WA-316-C3). Distance based on a cgMLST of 3140 genes and 540 accessory genes (wgMLST, 3680 genes) using the parameter “pairwise ignoring missing values” during calculation.

The wgMLST analysis showed that all four isolates clustered together with two to four allele differences (Figure 3). When comparing to all other available *L. anisa* genomes in NCBI, we found that the *L. anisa* Linanisette strain was the most closely-related genome (12 allele differences), whereas for the other 2 reference genomes, the number of allele differences was between 212 and 220.

### 3.3. Comparative Genomic Analysis

**Figure 4:**
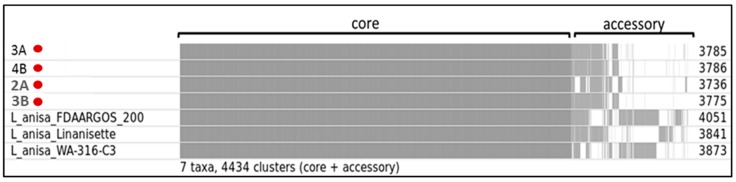
Roary matrix. Pan genome analysis of 7 *L. anisa* annotated genomes, four strains isolated from dental chairs and three reference genomes from NCBI database. Roary produced a gene presence/absence matrix with a total of 4434 protein-coding gene sequence clusters (grey color indicates presence, and white color indicates absence). The numbers on the right side indicate the number of clusters in each genome. The isolates from this study are marked in red.

The pan genome analysis showed that our isolates and the reference *L. anisa* strains shared the same core genome (Figure 4). However, regarding the accessory genes, strain 2A differed significantly from the other 3 isolates. The number of protein-coding gene sequence clusters was different in each isolate found in this study, ranging from 3736 to 3786. By looking at the accessory clusters, we can infer that the isolates from this study showed more similarities with the strain Linanisette compared to the other reference strains.

### 3.4. ARGs, VFs and Dot/Icm Effectors

**Figure 5:**
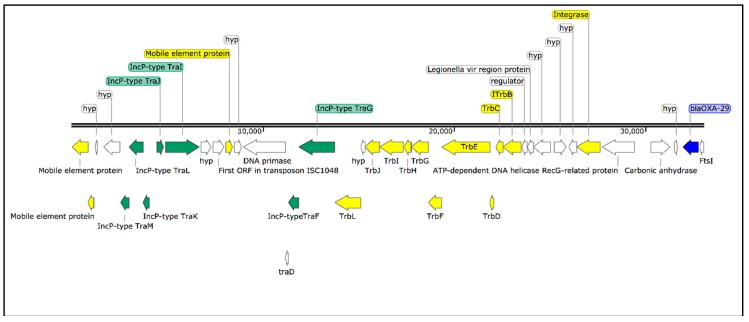
Schematic representation of *bla*_OXA-29_ resistance gene (blue) located downstream to an integrated plasmid (IncP type) (green) containing a transposon machinery (yellow).

RAST identified 8 different putative β-lactamases or β-lactamase-related genes. However, when we performed the analysis using ABRicate, we only found two previously described β-lactamase genes, the OXA-29 class D β-lactamase and the FEZ-1 metallo-β-lactamase, which were identified in all isolates. The analysis of the annotated hybrid assembly shows that the OXA-29 β-lactamase was located downstream to an integrated plasmid (IncP type) containing a transposon machinery (Figure 5). In addition, a mutation in the *gyrA* gene, leading to the amino acid change G81A, was detected in all isolates. Thirty-seven virulence genes were identified in all isolates, including the *mip* (macrophage infectivity potentiator) gene, *dot/icm* (delayed in organ trafficking/intracellular multiplication) genes, and several genes involved in cell motility (e.g., *fli* and *flg*) (Table 1). Regarding the Dot/Icm effectors repertoire, the isolates contained all known *Legionella anisa* effector proteins (*n* = 130), which are translocated into the host cells through the type-IV secretion system to alter the host-cell processes.

### 3.5. Plasmid Distribution

The hybrid assembly of isolate 3A produced three contigs of 4.2 Mbp (chromosome), 149 Kbp (plasmid p3A1), and 48 Kbp (plasmid p3A2) (Figure 5). Excluding the largest contig, representing the chromosome, we focused our analysis on the small contigs that most probably represented plasmid genomic sequences. A BLASTN analysis of p3A2 revealed *L. pneumophila* strain E9_O unnamed plasmid as the best match (99% coverage; 99% identity). On the other hand, p3A1 was not significantly similar to any plasmid sequence in the NCBI database (low query coverages between 10–40%), suggesting it is a new plasmid.

RAST annotation showed that p3A1 had 166 coding sequences (CDS), 6 repeat regions, and 81 proteins with functional assignment (e.g., IncF plasmid conjugative transfer proteins, cobalt- zinc-cadmium resistance proteins, and arsenic efflux pump protein). The analysis of p3A2 showed the presence of 53 CDS and 24 proteins with functional assignments (including IncF plasmid conjugative transfer proteins). The complete list of CDS belonging to the chromosome and to the plasmids is available as supplementary material.

**Figure 6:**
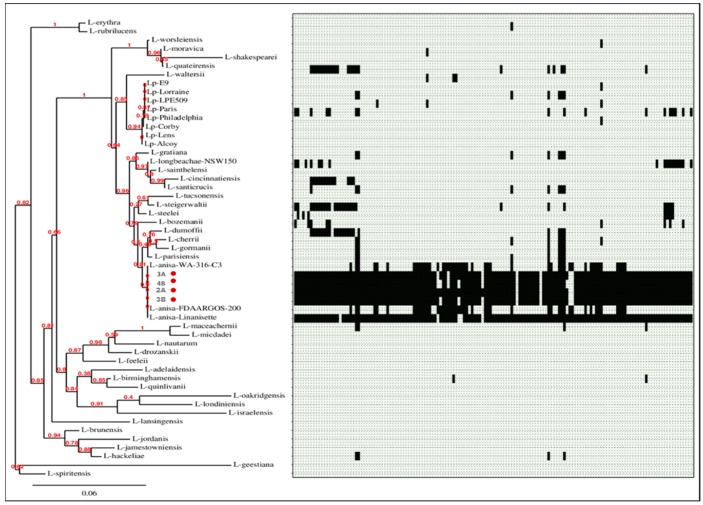
Phylogenetic tree of 53 *Legionella* genomes and SeqFindr presence/absence matrix of plasmid p3A1 (black color indicates presence, and light grey color indicates absence). The isolates from this study are marked in red.

**Figure 7:**
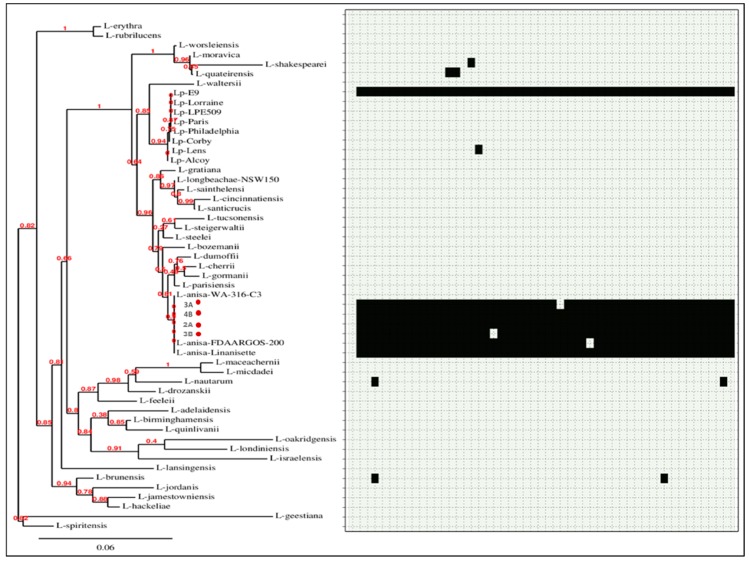
Phylogenetic tree of 53 *Legionella* genomes and SeqFindr presence/absence matrix of plasmid p2 (black color indicates presence, and light grey color indicates absence). The isolates from this study are marked in red.

SeqFindr analysis produced a presence/absence matrix showing the distribution of plasmid features among 53 *Legionella* genomes (49 plus 4 from this study) (Figure 6 and Figure 7). The analysis showed the presence of features of p3A1 in all our isolates and in the *L. anisa* strain Linanisette (Figure 5). Features of plasmid p3A2 were found in *L. pneumophila* strain E9_O and in all *L. anisa* genomes, except for *L. anisa* strain WA-316-C3.

## 4. Discussion

According to the Dutch regulation (Drinkwaterbesluit), which is comparable to the European Directive, drinking water must contain less than 0.1 CFU/mL of *Legionella* spp. Our study revealed *Legionella anisa* contamination in three out of four DCUs tested, with 100 CFU/mL, which represents a threat to the health of the patients and dental team. While the proportion of cases that are fatal tends to be much higher (30–40%) in nosocomial infections [26], nosocomial cases of LD represent a smaller percentage of reported cases of legionellosis than the community-acquired cases. For this reason, monitoring the hospital water-system, even in the absence of known cases of LD, is extremely important for the prevention of health-care associated infections (HCAIs). By combining classical culture methods with WGS-based investigation, this study revealed the presence of a unique *L. anisa* clone in several chairs of the same hospital dental ward. Long term persistence of highly similar isolates of *L. pneumophila* within several hospitals have been previously observed [27], underlining the ability of some strains to spread and persist in the hospital water systems. However, in our case, in order to overcome tap water contamination and to minimize risks for infections, all dental chairs and their pipelines belonging to that unit have been replaced. Further tests were performed after this intervention, and the results for *Legionella* were negative.

According to the wgMLST analysis, the *Legionella anisa* strain Linanisette represented the most closely related reference to our isolates. This strain was isolated from a respiratory sample using an amoebal co-culture procedure [28]. The pan genome analysis also confirmed the previous observation, suggesting a close genetic relatedness between this strain and our isolates. These observations, together with the detection of all 130 *L. anisa* Dot/Icm effectors, the *mip* gene, and several virulence factors, shows the potential of our 4 isolates to infect human macrophages. However, more studies are needed to confirm this capacity.

The pan genome analysis revealed all *L. anisa* strains had a conserved core genome, which is particularly relevant considering the different geographic location and source of isolation. In fact, while our strains, *L. anisa* strain FDAARGOS-200, and strain WA-316-C3 were isolated from hospital-related water in The Netherlands and USA, respectively), *L. anisa* strain Linanisette was found in a clinical sample. This observation suggests that this species has a very stable core genome. Despite the core genome, all strains showed substantial differences regarding the accessory genes. Indeed, *Legionella* genomes are characterized by a highly dynamic mobilome (species-specific phage-related elements, transposon, and plasmid) which allows the bacterium to rapidly adapt to environmental changes [29].

Regarding ARGs, the OXA-29 class D β-lactamase and FEZ-1 metallo-β-lactamase determinants were identified in the chromosome of all isolates. In particular, as mentioned before for the isolate 3A, a *bla*_OXA-29_ gene was located downstream to an integrated plasmid (IncP type). BLASTN analysis of *bla*_OXA-29_ showed the presence of this gene in the genome of *L. gormanii*, as previously described [30], in three *L. pneumophila* plasmids (GenBank: CP021284.1; GenBank: CP011106.1; GenBank: FQ958212.1) and in one plasmid belonging to *L. sainthelensi* (GenBank: CP025492.1). These observations underline the high rate of interspecies gene transfer among *Legionella* spp. and the risk of potential intergenus transferring to more pathogenic bacteria, as has occurred in the past [31]. This is especially worrisome in a hospital setting, where the biofilm formation in the water pipelines and a higher antibiotic selective pressure raises the chance for antibiotic resistance transmission [32]. However, the importance of this beta-lactamase in the genomes of *Legionella* spp. strains remains to be elucidated, since beta-lactams are not used to treat legionellosis.

The acquisition of resistance through point mutations is particularly important in *Legionella* species [21]. The amino acid change G81A, which is linked to fluoroquinolones resistance, has been previously described in one *in vitro* mutant *L. pneumophila* strain [20]. In this study, all isolates had MICs ≤ 0.5 and ≤ 0.25 to moxifloxacin and levofloxacin respectively, which confirms that accumulative substitutions are needed to confer resistance [20]. The antibiotic selective pressure expected in the hospital environment is higher than in other settings; further analysis of the presence of antibiotic residues in dental water pipelines would help to elucidate the forces that promote the evolution of antibiotic resistance.

By combining ONT long reads and short reads from Illumina, we succeeded in obtaining two contigs representing two complete and circular plasmid sequences, improving the quality of ARGs and VFs analyses. The annotation of these plasmid sequences showed the presence of several proteins related to IncF-type and IncP-type conjugative systems, together with numerous proteins associated with heavy-metal resistance. In addition, the presence of plasmid p3A2 in our *L. anisa* isolates and in *L. pneumophila* strain E9_O may suggest that interspecies horizontal gene transfer (HGT) occurs between both species.

## 5. Conclusions

Combining standard culture method with WGS allowed the identification of a unique clone of *L. anisa* in several DCUs in the same hospital dental ward. This may indicate there was a common contamination source in the dental unit waterlines, which was resolved by replacing the chairs and main pipeline of the unit. Deep environmental sampling and WGS-based typing methods are relevant to map the occurrence of *Legionella* spp. in the hospital environment for future reference in nosocomial LD cases. Our results revealed tap water contamination in direct contact with patients and the usefulness of WGS to investigate bacterial molecular epidemiology.

## Figures and Tables

**Table 1:** Virulence factors detected in all 4 isolates.

Product/Function	Gene	Average Coverage (%)	Average Identity (%)
Macrophage infectivity potentiator	*mip*	98.59	78.75
Dot/Icm secretion system	*ceg*5	100	78.93
	*dot*B	96.31	76.98
	*dot*C	88.38	76.78
	*dot*D	98.78	77.5
	*icm*B/*dot*O	99.5	76.26
	*icm*D/*dot*P	96.1	84.87
	*icm*D/*dot*P	92.2	78.46
	*icm*J/*dot*N	98.6	76.92
	*icm*L/*dot*I	99.84	79.62
	*icm*O/*dot*L	99.7	78
	*icm*S	100	79.13
	*icm*T	98.47	75.77
	*icm*W	100	77.85
	*lem*8	96.35	81.57
	*lpg*0181	100	80.7
	*lpg*0260	84.46	75.29
	*lpg*2359	97.75	75.78
	*lpg*2372	100	94.65
	*lpg*2539	89.46	77.19
	*lpg*2552	98.92	86.75
	*rav*L	83.33	76.24
Motility	*fle*R/*flr*C	96.48	75.38
	*flg*C	99.76	75
	*flg*I	94.01	76.12
	*flh*A	99.71	75.62
	*fli*G	96.36	76.1
	*fli*P	89.6	78.72
	*pil*T	97.29	79.05
Others	*ccm*C	99.37	75.97
	*enh*A	89.07	75.65
	*htp*B	99.88	85.07
	*ira*A	100	75.34
	*lsp*E	99.66	78.49
	*lsp*G	97.64	79.18
	*pht*A	98.29	76.39
	*sod*B	99.66	76.74

## Supplementary Materials

The following are available online at http://www.mdpi.com/2076-2607/6/3/71/s1, Table S1: Annotations of the genomic sequences, Table S2: List of accession numbers used for comparison purposes.
